# Case of Puerperal Convulsions, Treated with Chloroform

**Published:** 1848-09

**Authors:** James P. White

**Affiliations:** Bufealo


Buffalo, August, 1848.

---

ART. II.—*Case of Puerperal Convulsions, treated with Chloroform.* By James P. White, M. D.

*July* 18*th* 1848. Was requested by Dr. Devening to see Mrs. Brady, then in labor, and suffering from a violent attack of puerperal convulsions. I found an Irish woman, of full habit, in her third pregnancy, the two previous labors having been favorable. Dr. D. had been with her but a short time; the convulsive paroxysms succeeding each other with great rapidity; the face flushed and pulse full. He had bled her twenty or twenty-four ounces with partial relief. As the pulse continued full, and the muscular efforts during the fits were violent, I deemed it prudent to repeat the bleeding, which was done to about the previous amount. On examination per vaginam, the breech was found occupying the superior strait, the soft parts dilatable, and the pelvis roomy. As the convulsions continued with but slight diminution, and the parts were favorable, though the pains were slight, it was deemed prudent to proceed without delay to deliver the child. To secure the tonic contraction of the uterus, and guard against hemorrhage, two drachms of the wine of ergot were given. It occurred to me that chloroform might assist in controlling the woman, and lessening the agitation while making the attempt to bring down the child. Dr. D. accordingly applied some of Chilton's preparation to the nostrils, whilst I introduced my fingers into the vagina, and hooked them into the groin of the fœtus.

The muscular agitation, which before could not be controlled, now subsided, and delivery was effected without difficulty; the uterine contraction continuing to be moderate. After applying such restoratives as were requisite to recover the child from a state of partial asphyxia, I applied a ligature to the cord, and laid it upon the bed. By placing the hand upon the abdomen, it was found to be still distended. Upon the re-introduction of the finger into the vagina, it came in contact with the arm of a second child, which I proceeded to turn and deliver, the patient inhaling the chloroform, and remaining quiet, with tolerably firm contraction of the uterus upon the hand during the operation. The double placenta and membranes were very soon pushed down into the vagina by the tonic contraction of the uterus, and were removed by Dr. Devening. Very little hemorrhage followed, and the proper bandage being applied, the patient was carefully laid back upon the bed. During the entire period succeeding the application of the anæsthetic agent, the woman was unconscious and calm, but at no time did we carry it so far as to produce stertorous breathing.

21*st.* The patient comfortable, and consciousness entirely restored. She has no recollection of any thing which occurred during her labor after being seized with convulsions. The children are of good size, and doing well.

25*th.* From Dr. Devening I learn that the woman has so far recovered as to resume her usual domestic duties.

I have been thus full in the description of this case, from the fact that Chloroform was resorted to. On the introduction of any new medicinal agent, it ordinarily occurs, that a part of the profession become advocates for its indiscriminate use; while, for no better apparent reasons than the love of opposition, or distaste for innovation, another party is formed who perseveringly contest its claims to favor. The recent controversies regarding the propriety of the use of the agent in question, are a sufficient evidence of the truth of this remark. On the one hand we find Dr. Simpson giving it to all parturient females, saying that, "in midwifery, most, or all of my brethren in Edinburgh employ it *constantly*;" that "no accidents have as yet happened, though several hundred thousands must have already been under its influence;" and he most complacently adds:

"Those who most bitterly oppose its obstetric employment now, will be, ten or twenty years hence, amazed at their own professional cruelty." On the other hand, we find the scarcely less celebrated American Obstetrician, Dr. Meigs, a man, who, like Dr. Simpson, is usually found occupying a partizan position in regard to any novelty, in this instance strenuously opposing its use under all circumstances. He has not exhibited it in any case, nor has he any intention of so doing, remarking in his own peculiar style, "I have been accustomed to look upon the sensation of pain in labor, as the physiological relation of the power of force; and notwithstanding, I have seen so many women in the throes of labor, I have always regarded a labor pain as a most desirable, salutary, and conservative manifestation of life-force. I have found that women, provided they are sustained by cheering counsel and promises, and carefully freed from the distressing element of terror, could in general be made to endure, without great complaint, those labor-pains which the friends of anæsthesia desire so earnestly to abolish and nullify, for all the fair daughters of Eve. Dr. M. believes pain not only necessary, (and rightly as we think in many instances;) but desirable as a guide in obstetric operations; and that mere exemption from pain for a short time, would not justify him "in casting away his safest and most trustworthy diagnosis."

Opposed, as we always are, to "meddlesome midwifery," we can have no doubt that any interference with the process of healthy labor is sure to be followed, sooner or later, by injurious, if not fatal consequences. The advocates for the use of Chloroform do not pretend, that under ordinary circumstances, it does more than merely relieve physical pain. Now if this interference with this hitherto invariable accompaniment of natural labor can be shown to be entirely safe, then it may be well to take the question of the propriety of its uniform use, into serious consideration. A careful reference to the cases reported in the various American and European Journals, will be sufficient, at least, to throw great doubt on this point. For the present, therefore, we are prepared to conclude with Baron Dubois, that: "Its use in midwifery should be restrained to a limited number of cases, the nature of which, ulterior experience will better allow us to determine." It seems to us that anæsthetic agents may be applicable in puerperal convulsions, if, after proper depletion, the muscular contractions continue violent, and especially if turning or other interference become necessary for the relief of the patient. The woman is already unconscious, and you do not, as is the case where unconsciousness is purposely induced, deprive yourself of a valuable guide in the application and use of instruments, On the contrary, if it should produce quietude, you secure an almost indispensable prerequisite to the safe performance of any operation at, or above the brim of the pelvis. Whether in the case of Mrs. B. the quietude was the result of the inhalation of the Chloroform, or of the copious bleedings, without which, in this case, as we think, it would have been unsafe to resort to it, it may be difficult to determine. It is certainly a most *remarkable* instance of coincidence, if the subsidence of the eclampsia were *not* the result of the *combined* effect of the Chloroform and bleeding; and, in our opinion, the result can scarcely be attributed to the single action of the latter.

A careful discrimination in the selection of cases, and a resort to it only with the proper precautions, may, by securing a number of well-attested observations, place in the hands of the Obstetrician an important auxiliary, though none but an enthusiast will look for a panacea.

It is in the hope of contributing in some slight degree to a more rational and judicious selection of cases for the exhibition of anæsthetic agents that this case been furnished.

